# “It seems to have drawn us closer”: Participant Reflections on Digital Advance Care Planning

**DOI:** 10.1093/geroni/igaf122.4055

**Published:** 2025-12-31

**Authors:** Sharon Bigger, Jordana Clayton, Sara Bybee, Nancy Aruscavage, Eli Iacob, Rebecca Utz, Kara Dassel

**Affiliations:** University of Utah, Asheville, North Carolina, United States; University of Utah, Salt Lake City, Utah, United States; University of Utah, Salt Lake City, Utah, United States; University of Utah College of Nursing, Salt Lake City, Utah, United States; University of Utah, Salt Lake City, Utah, United States; University of Utah, Salt Lake City, Utah, United States; University of Utah, Salt Lake City, Utah, United States

## Abstract

Persons living with dementia (PLWD) and their care partners (CP) experience improved outcomes when engaging in advance care planning (ACP). We explored participant reflections on a web-based intervention for ACP in dementia. A thematic analysis was conducted on open-text feedback data from N = 47 dyads (PLWD and CP). Two coders analyzed data independently, resolved discrepancies, and collaborated with the research team to determine themes. The sample was mostly female (54%), white (86%), and aged 26 to 93. Among care recipients, 67% had mild cognitive impairment or dementia; the remainder reported early warning signs of Alzheimer’s disease. Three themes included: (1) Dynamic nature of ACP in dementia, (2) Cognitive issues, and (3) Emotional issues. Findings revealed that dyads (PLWD and CP) used the web-based intervention for ongoing, dynamic ACP despite cognitive issues and emotional issues. For example, despite PLWD “needing questions clarified” and having “frustration” and “overwhelm,” CPs stated, “It seems to have drawn us closer,” “These are important and necessary plans to make, but it is sad to go through this process,” and “Even though (we) have already completed our directives…this…helped to have additional considerations for our end-of-life plan…” These findings support a Delphi expert panel definition of ACP in dementia as a continued process with family over time that is adapted to the PLWD’s capacity. We add that ACP remains beneficial even in the face of cognitive and emotional challenges. Researchers and clinicians should acknowledge cognitive and emotional issues for persons engaging in dynamic ACP in dementia.

